# Metabolomics combined with network pharmacology reveals the potential development value of *Campanumoea javanica* Bl. and its metabolite differences with Codonopsis Radix

**DOI:** 10.1186/s12870-024-05401-0

**Published:** 2024-07-18

**Authors:** Jie Peng, Sha Liu, Xuanlin Wu, Shuo Li, Jian Xie, Yong Wang, Qiuyang Yao, Faming Wu, Delin Zhang

**Affiliations:** 1 Clinical Pharmacy, Affiliated Hospital of Zunyi Medical University, Zunyi, 563003 China; 2 School of Pharmacy, Zunyi Medical University, Zunyi, 563000 China; 3 College of Pharmacy, Gansu University of Chinese Medicine, Lanzhou, 730000 China; 4 Guizhou Medical and Health Industry Research Institute, Zunyi, 563000 China; 5 State Key Laboratory of Functions and Applications of Medicinal Plants, Guizhou Medical University, Guiyang, 550000 China

**Keywords:** *Campanumoea javanica* Bl., Codonopsis radix, Metabolite, Widely targeted metabolomics, Network pharmacology

## Abstract

*Campanumoea javanica* Bl. (CJ) traditionally used in Southwestern China, is now widely consumed as a health food across the nation. Due to its similar efficacy to Codonopsis Radix (CR) and their shared botanical family, CJ is often used as a substitute for CR. According to the Chinese Pharmacopoeia, *Codonopsis pilosula* var. *modesta* (Nannf.) L.T. Shen (CPM), *Codonopsis pilosula* (Franch.) Nannf. (CP), and *Codonopsis tangshen* Oliv. (CT) are the primary sources of CR. However, details on the differences in composition, effectiveness, and compositional between CJ and CR are still limited. Besides, there is little evidence to support the application of CJ as a drug. In this study, we employed widely targeted metabolomics, network pharmacology analysis, and molecular docking to explore the disparities in metabolite profiles between CJ and CR and to predict the pharmacological mechanisms of the dominant differential metabolites of CJ and their potential medicinal applications. The widely targeted metabolomics results indicated that 1,076, 1,102, 1,102, and 1,093 compounds, most phenolic acids, lipids, amino acids, and flavonoids, were characterized in CJ, CPM, CP, and CT, respectively. There were an average of 1061 shared compounds in CJ and CRs, with 95.07% similarity in metabolic profiles. Most of the metabolites in CJ were previously unreported. Twelve of the seventeen dominant metabolites found in CJ were directly associated with treating cancer and lactation, similar to the traditional medicinal efficacy. The molecular docking results showed that the dominant metabolites of CJ had good docking activity with the core targets PIK3R1, PIK3CA, ESR1, HSP90AA1, EGFR, and AKT1. This study provides a scientific basis for understanding the similarities and differences between CJ and CR at the metabolome level, offering a theoretical foundation for developing innovative medications from CJ. Additionally, it significantly enhances the metabolite databases for both CJ and CR.

## Introduction

*Campanumoea javanica* Bl. (CJ), is known as “Tudangshen” in Chinese and has been utilized in the national medicine system of China for hundreds of years. CJ is a perennial herbaceous dicotyledonous plant widely distributed in the tropical and subtropical regions of eastern Asia. It belongs to the genus *Campanumoea*, which is part of the family Campanulaceae. CJ is not only a commonly used herb medicine in the Han nationality but also nine minority habitual drugs including Yi, Miao, and Yao. CJ was first recorded in the Qing Dynasty’s *An Illustrated Book of Plants* [1] and later recorded in the *Chinese Pharmacopoeia* [2], *Guizhou Quality Standards for Traditional Chinese Medicines and Ethnic Herbs* [3], *Guizhou Standards for the Preparation of Traditional Chinese Medicinal Pieces* [4], and *Standards for the Preparation of Traditional Chinese Medicinal Pieces of the Guangxi Zhuang Autonomous Region* [5]. CJ has been used in Chinese medicine to treat internal injuries due to pulmonary asthenia, spleen deficiency diarrhea, lactation, and infantile enuresis [6]. To date, CJ has been produced into a variety of Chinese patent drugs for national use, such as Benglu Pill, Baidai Pill, Qinghuoyangyuan Capsules, Jinwugutong Capsules, Hulisan Capsules. The Hulisan Capsules is a famous bloodstasis type of Chinese patent drug in Yunnan, selling more than 158 million yuan in 2014 [7–10]. In addition, CJ is also an important edible plant and is often used as a medicinal dietary ingredient [11]. These biological activities were attributed to its high content of polysaccharides [12]. CJ is naturally distributed widely in Southern China, including Guizhou, Sichuan, Yunnan, and Guangxi provinces [13].

Codonopsis Radix (CR) is a famous traditional Chinese medicine with a long history of use in pharmaceuticals and edibles. It is one of the most popular herbs of traditional Chinese medicine today and is well regarded by the public as a nourishing drug. The CJ and CR demonstrate a certain level of similarity in efficacy and belong to the same family. Consequently, the CJ is often used as a substitute for CR. In fact, the genus of CJ is *Campanumoea* within the family Campanulaceae, while CR belongs to the genus *Codonopsis* in the same family. Moreover, CJ and CR are each unique in terms of their efficacy; for example, there is a significant difference between them in the treatment of pediatric enuresis and lactation disorders. The confused application of the two might result in dangerous effects, including medical mishaps. Unfortunately, although a few studies have identified them, effective methods to distinguish them are still lacking. It is difficult to accurately identify its varieties in the process of market circulation. CJ and CR are often mixed into one thing, which causes unnecessary difficulties in the treatment of clinical diseases in Chinese medicine. Up until now, there have been only a few phytochemical studies on CJ. Zhang et al. isolated 6 compounds from CJ, including two lignans (syringin and dangshen glycosides I) and two flavonoids (5-hydroxy-4’, 6, 7-trimethoxyflavone, and 5-hydroxy-4’, 7-dimethoxyflavone) [14]. In addition, zanthocapensol, dangshen glycosides II, and 2 anti-angiogenic active ingredients (4E, 8E, 12E-triene-10-yne-1, 6, 7-tetradecanetriol, and 9-(2-tetrahydropyran)-8E-en-4, 6-diyne-3-nonanol) were isolated from CJ [15]. According to the available literature, more than 50 compounds including glycosides, alkynes, flavonoids, and phenylpropanoid compounds have been isolated from CJ [16–18].

Currently, little is known about the phytochemical distinctions between CJ and CR, as well as the potential medical development value of CJ. In this study, ultra-performance liquid chromatography-electrospray ionization-tandem mass spectrometry (UPLC-ESI-MS/MS) integrating chemometrics was applied for metabolite profiling and discrimination of CJ and CR. Targeted metabolomics analysis was used to reveal metabolic differences between CJ and CR in metabolism and to screen for dominant differential metabolites of CJ. The network pharmacology was employed to predict the pharmacological effects of dominant differential metabolites and explore the potential medicinal value of CJ. Furthermore, we used molecular docking techniques to dock their core targets with the active ingredients to confirm the reliability of the network pharmacological predictions. This is a comprehensive investigation of CJ phytochemistry and compares the chemical findings of CJ and CR, providing a better understanding of the differences between CJ and CR, and giving references for the quality control of herbs and the medicinal development of CJ.

## Materials and methods

### Sample collection

**Fig. 1:**
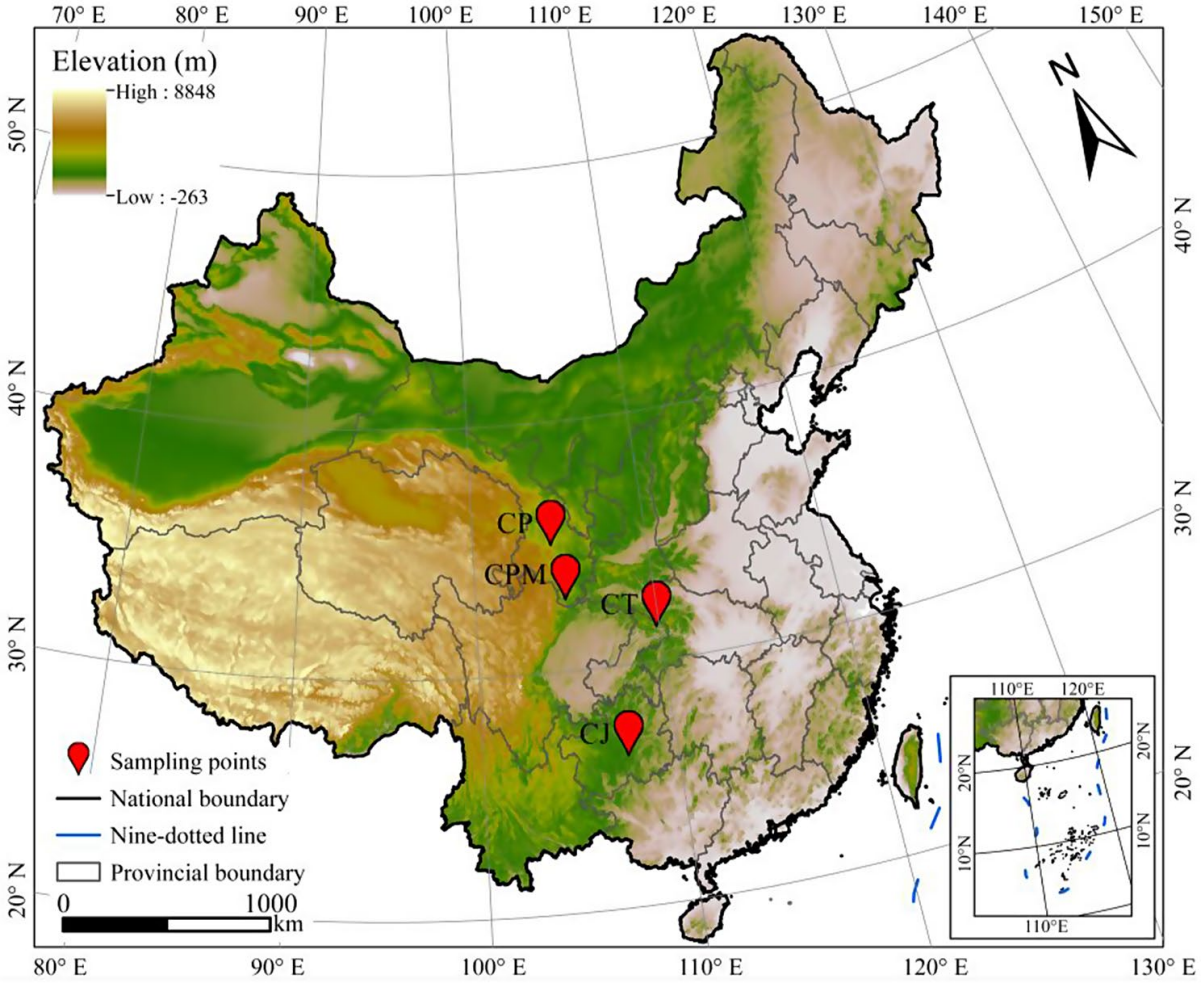
The regional distribution of samples

From August 2021 to October 2021, a total of twelve samples were collected in the production regions. CJ samples were collected from Guiding County, Guizhou province, while *Codonopsis pilosula* (Franch.) Nannf. (CP), *Codonopsis pilosula* var. *modesta* (Nannf.) L.T. Shen (CPM), and *Codonopsis tangshen* Oliv. (CT) were obtained from Weiyuan County (Gansu province), Wen County (Gansu province), and Wuxi County (Chongqing municipality), respectively (Fig. 1). Professor Faming Wu, College of Pharmacy, Zunyi Medical University, identified CJ, CT, CP, and CPM as the roots of *Campanumoea javanica* Bl., *Codonopsis tangshen* Oliv., *Codonopsis pilosula* (Franch.) Nannf., and *Codonopsis pilosula* var. *modesta* (Nannf.) L.T. Shen, respectively. Each sample weighed between 1,000 and 1,500 g, and three samples was collected from each region. The samples were promptly brought to the laboratory, washed, and separated into aboveground and subsurface sections that were packed in self-sealing bags. For metabolite extraction, the subterranean section was held at -80 °C. All samples are stored in the laboratory of Zunyi Medical University.

### Sample preparation

Sample preparation was conducted with the method of Zhang KaiXian [19]. In a Scientz-100 F freeze-dryer (Scientz, China), fresh CJ and CR samples were vacuum freeze-dried. Subsequently, they were ground into a powder using a mixer (MM 400, Retsch, Germany) at 30 Hz for 1.5-2 min. Approximately 50 mg of the sample was dissolved in 1.2 mL of 70% methanol extract (Merck, Germany), which was then vortexed (IKA VORTEX GENIUS 3, VG 3 S25) every 30 min for 30 s for a total of 6 times. The sample was placed at 4℃ overnight. Afterward, it underwent a 10-minute centrifugation at 12,000 rpm (Allegra 64R, Beckman, USA). Following the centrifugation, the supernatant was filtered using a 0.22-µm microporous membrane and then transferred to a vial for analysis using ultra-performance liquid chromatography-electrospray ionization-tandem mass spectrometry (UPLC-ESI-MS/MS) analysis.

### UPLC-ESI-MS/MS analysis

Broadly targeted metabolomics employed AB sciex TripleTOF660 high resolution mass spectrometry for qualitative detection of mixed samples, followed by AB sciex6500 QTRAP quantification, combining the benefits of untargeted and targeted metabolomics to achieve precise qualification via high resolution mass spectrometry. Using UPLC-ESI-MS/MS for broadly targeted metabolomics, the sample extracts were analysed [20]. Chromatographic separation was performed on a Nexera X2 UPLC machine (Shimadzu, Kyoto, Japan) utilising an Agilent SB-C18 column (1.8 μm, 2.1 mm, 100 mm; Agilent, Foster City, CA, USA). The mobile phase consisted of acetonitrile combined with 0.1% formic acid (solvent B) and purified water containing 0.1% formic acid (solvent A). 0–9 min (95%→5% A), 9–10 min (5% A), 10–11.1 min (5%→95% A), and 11.1–14 min (95% A) comprised the gradient elution program. The injection volume was 2 µL, the flow rate was 0.35 mL/min, and the temperature setting for the column oven was 40 °C.

The alternative technique for injecting the effluent was a triple quadrupole-linear ion trap mass spectrometer (6500 Q-TRAP UPLC-MS/MS; Applied Biosystems, Framingham, MA, USA) equipped with an ESI Turbo Ion-Spray interface. The ESI ion source was operated with the following parameters: turbo spray ion source; 550 °C temperature; 5,500 V spray voltage (positive ion mode)/-4,500 V spray voltage (negative ion mode); gas I and gas II pressures of 50 and 60 psi respectively; curtain gas pressure of 25.0 psi; and collision-activated dissociation set to high. Triple quadrupole and linear ion trap modes were utilised for the calibration of the instrument and mass, using solutions of 10 and 100 µmol/L polypropylene glycol, respectively. Triple quadrupole pictures were obtained using the multiple reaction monitoring (MRM) assays with the collision gas (nitrogen) set to medium. For each MRM transition, the collision energy and delustering potential were tuned to maximise efficiency. Based on the metabolites that eluted at that time, a predetermined set of MRM transitions was looked for throughout each session. To gather and examine mass spectra, AB Sciex, Foster City, CA, USA, provided Analyst v1.6.3.

The metabolites in the project samples were subjected to qualitative and quantitative mass spectrometry analysis utilizing the Metware Biotechnology Co., Ltd. (MWDB), KEGG compound database, and MRM. Metabolite identification was conducted utilizing retention time (RT), MS2 fragmentation, metabolite precise mass, and MS2 fragment isotopic distribution. The secondary spectrum and RT of metabolites in the project samples are intelligently matched one by one with the secondary spectrum and RT of the company’s database through the company’s self-developed intelligent secondary spectrum matching method with the MS and MS2 tolerances set to 20 ppm and 20 ppm, respectively, and the RT tolerance of 0.2 min.

To ensure the precision of the metabolite annotations, interference signals were eliminated at the outset of each study. These signals included the repeated signals of K^+^, Na^+^, and NH^4+^ ions, fragment ions, and the isotope signal. We used Multi Quant v3.0.2 (AB Sciex) for MRM-based measurement of metabolites. In MRM mode, interference is initially eliminated by screening the precursor ions (parent ions) of the target substances through the four-stage rod and excluding the ions corresponding to other molecular weight substances. The collision chamber induces the precursor ions to ionize and break, resulting in the formation of numerous fragment ions. These fragment ions are subsequently filtered through the triple four-stage rod in order to select a characteristic fragment ion necessary for excluding the interference of non-target substances. Once the metabolite spectra of various samples were acquired, the peak area of each peak in the mass spectra was integrated. Subsequently, the mass spectra of identical metabolites in different samples were adjusted to account for the integration of the peaks [21–23].

### Network pharmacology

Using the PubChem database (https://pubchem.ncbi.nlm.nih.gov/), the canonical SMILES of the prominent differential metabolites were searched. The relevant target proteins were then predicted using the Swiss Target Prediction database (http://www.swisstargetprediction.ch/). Once the pertinent target proteins were sorted out and the repeated targets were removed, the targets of the dominating differential metabolites were found [24].

A network was constructed and visualized with default parameters and similarity thresholds using STRING (http://string-db.Org, high confidence > 0.9) and Cytoscape v3.9.1 [25, 26]. The Kyoto Encyclopaedia of Genes and Genomes (KEGG, https://www.genome.jp/kegg/), Gene Ontology (GO, http://geneontology.org/) functional enrichment, and disease enrichment analyses were carried out using the David database (https://david.ncifcrf.gov/) (*P* < 0.01).

### Molecular docking

The PPI results were imported into Cytoscape 3.9.1 software in TSV text format, and the topological attributes of the results were analyzed by using the “Network Analyzer” function to obtain four topological parameters (Degree centrality, Betweenness centrality, Closeness centrality, Eigenvector centrality) [27].

The active ingredients screened from CJ were validated through molecular docking with core targets using Autodock software. The 3D structures of the ligand active ingredients were obtained in mol 2 format from Zinc (http://zinc.docking.org/substances/home/) and PubChem databases, while the three-dimensional structures of target receptor proteins were retrieved from the protein database (RCSB) (http://www.rcsb.org/), then PDB format files were downloaded. Subsequently, water molecules and excess inactive ligands were removed from the proteins using PyMOL 2.4.1 software. The processed results were imported into AutoDock Tools 1.5.6 software for hydrogenation, charge addition, and output in pdbqt format [28].

The receptor and ligand pdbqt formats were imported into AutoDock to define the molecular docking range. By setting the target protein as the center of the grid, the center coordinates (center 3.48/2.398/11.641) and box size (size 66/52/62) parameters were adjusted to ensure complete coverage of the protein by the docking box.

In AutoDock, molecular docking involves identifying protein macromolecules, inserting small drug molecules, and setting up operational methods and docking parameters. The pdbqt format is utilized for calculating the minimum binding energy and visualizing the results with Origin software. OpenBabelGUI is used to convert the combined pdbqt format to PDB format, and the docking results are visualized using PyMOL 2.4.1 software [29].

### Statistical analysis

The raw data was arranged using Excel 365 for Microsoft Office. Principal component analysis, Pearson correlation analysis, hierarchical cluster analysis, *K*-means cluster analysis, orthogonal partial least squares-discriminant analysis (OPLS-DA). Metabolites with variable importance in projection (VIP, OPLS-DA) and |log2fold change|>1 were considered differential metabolites (DMs) [30].

## Results and analysis

### Widely targeted metabolomics analysis

#### The differences in metabolite accumulation between *Campanumoea javanica* bl. And Codonopsis Radix

**Fig. 2:**
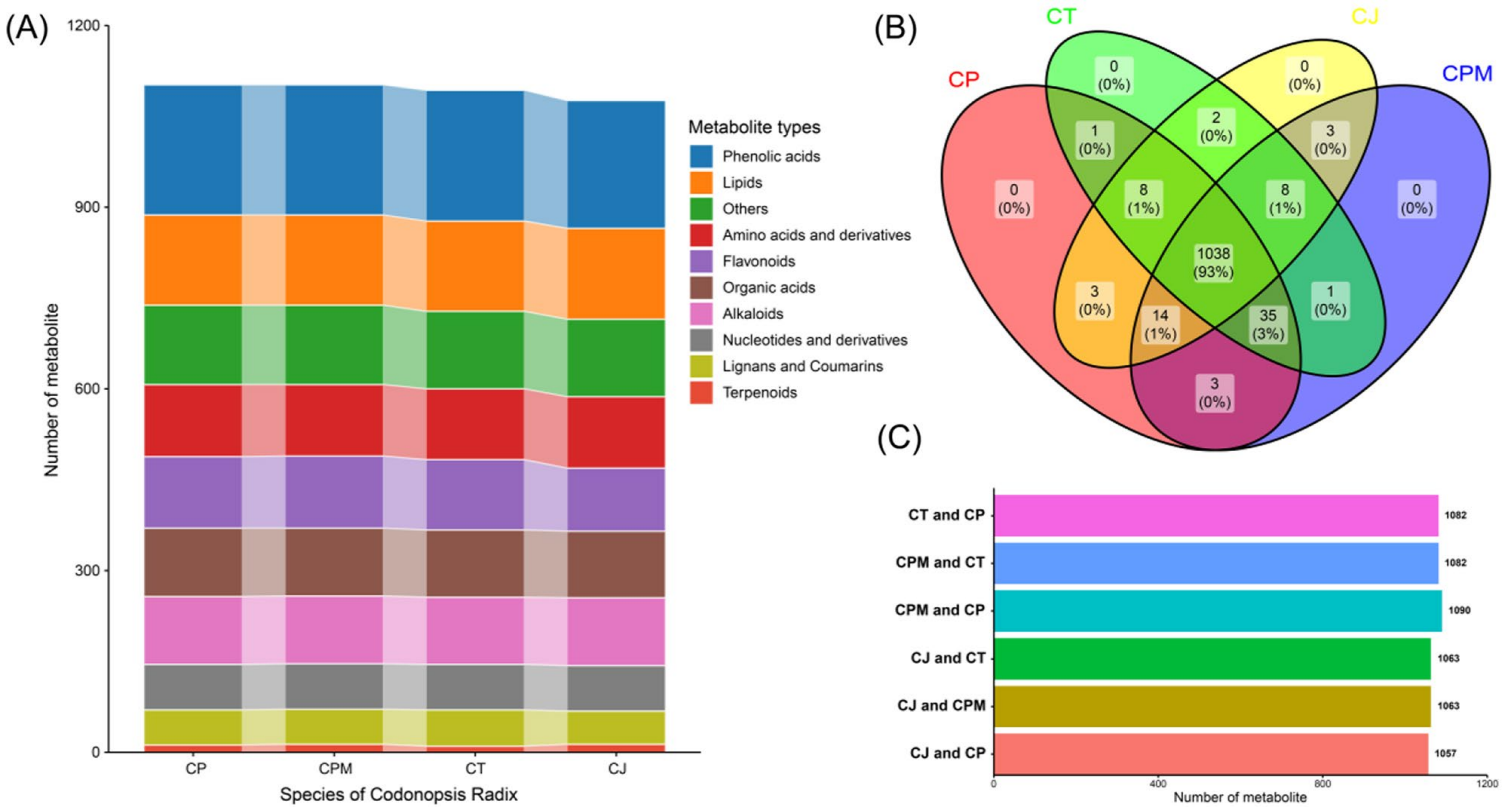
Analysis of differences in metabolite accumulation between Codonopsis Radix and *Campanumoea javanica* Bl. (**A**) Type distribution of metabolites; (**B**) Venn diagram of metabolites; (**C**) The quantity of metabolites shared by different samples

To learn more about the variations in metabolite accumulation between CJ and CR, we analyzed the metabolites using UPLC-ESI-MS/MS. In CJ and CR, about 1,116 metabolites were discovered and identified, categorized into ten distinct chemical types (Fig. 2A). The number of metabolites was 1,102 (CPM) = 1,102 (CP) > 1,093 (CT) > 1,076 (CJ). Phenolic acids were the most prevalent class (214–216). The remaining classes were lipids (145–150), flavonoids (104–119), amino acids and their derivatives (117–119), organic acids (110–113), alkaloids (111–112), derivatives (75), terpenoids (10–13), lignans and coumarins (55–60), others (128–131).

A Venn diagram was applied to analyze the differences in metabolite classes between CJ and CR aiming better to understand the variations in metabolites (Fig. 2B). The results showed that 1038 metabolites were shared between CJ and three CRs, including phenolic acids, lipids, amino acids and derivatives, alkaloids, organic acids, flavonoids, nucleotides and derivatives, lignans, coumarins, and terpenoids. The numbers of metabolites in each category were 204, 146, 116, 124, 108, 105, 98, 75, 52, and 10, respectively. Compared with CJ, 17 or 26 more compounds were identified in CPM, CT, and CP, mainly flavonoids and phenolic acids.

CJ shared 1057, 1063, and 1063 metabolites with CP, CT, and CPM, respectively (mean 1061), and the average similarity of metabolic profiles was 95.07%, and no unique compounds were found in CJ. The shared metabolites among three CRs were 1082–1090 (mean 1085), and the average similarity of metabolic profiles was 97.19%. The results indicated that CJ and the three CRs had remarkably similar metabolic profiles, thereby providing material basis supporting the potential use of CJ as a substitute for CR (Fig. 2C).

**Fig. 3:**
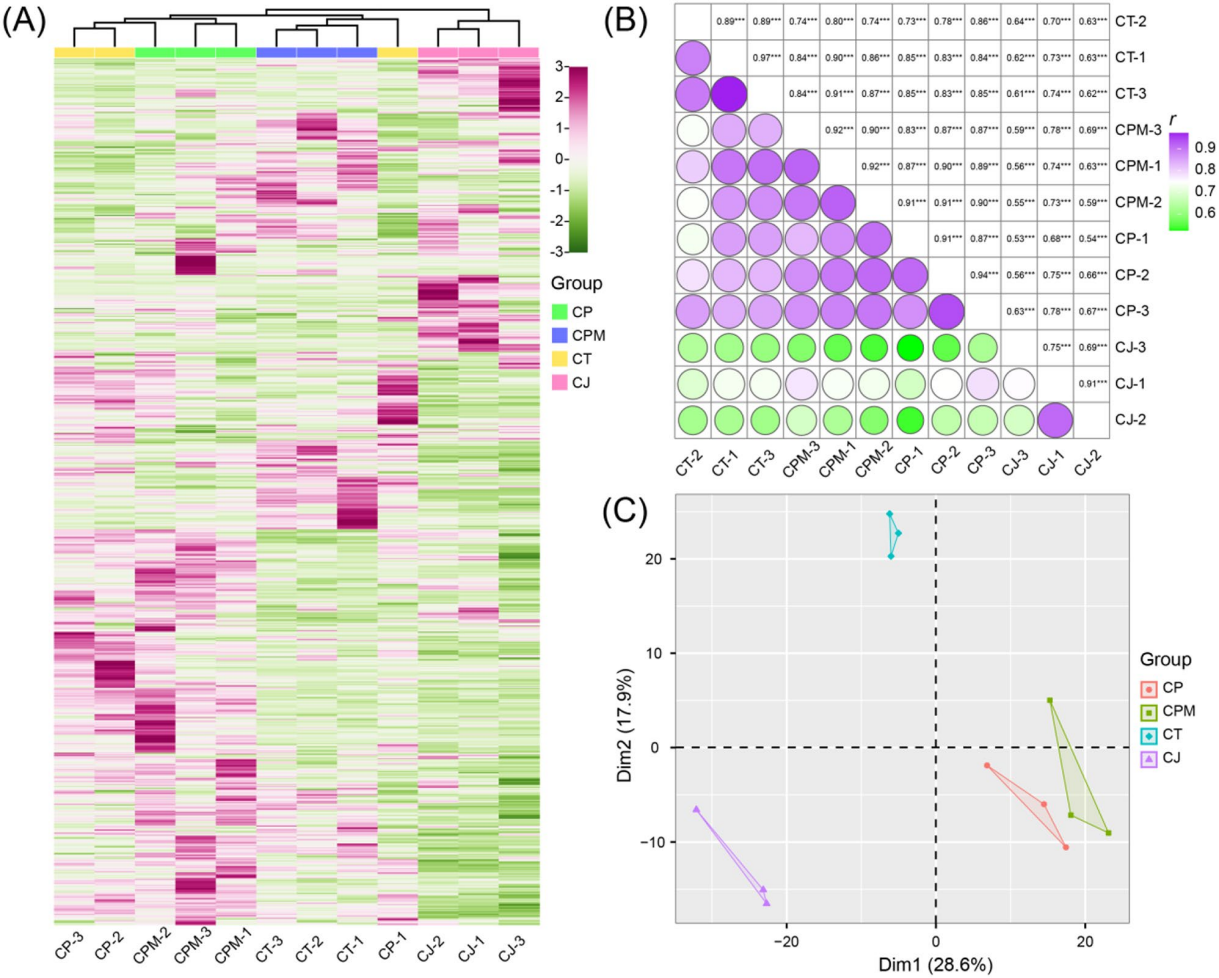
Multivariate statistical analysis of different metabolites. (**A**) Hierarchical cluster analysis of Codonopsis Radix and *Campanumoea javanica* Bl. metabolites; (**B**) Pearson correlation analysis of Codonopsis Radix and *Campanumoea javanica* Bl. metabolites; (**C**) Principal component analysis of Codonopsis Radix and *Campanumoea javanica* Bl. metabolites

We performed principal component analysis, Pearson correlation analysis, and hierarchical cluster analysis to further assess fundamental characteristics and differences of metabolites between CJ and CR. According to the hierarchical cluster analysis results, CP, CPM, and CT metabolites were grouped in one category, whereas CJ metabolites were classified separately. It suggests that CP, CPM, and CT metabolites exhibit distinct accumulation characteristics compared to CJ (Fig. 3A). Pearson correlation analysis showed that the correlation coefficient of samples in the group was greater than that of species. So, the reliability of the test data was high, and the results of subsequent analyses were credible (Fig. 3B). Dim1 and Dim2 accounted for 28.6% and 17.9% of the overall variance, respectively, according to principal component analysis. Dim1 significantly separated CJ and CT (CP, CPM), and Dim2 significantly separated CT from the other three groups (Fig. 3C). Thus, the results of the analysis indicated that the metabolic profiles of CJ are distinct from those of CP, CT, and CPM, with some differences in content profiles.

**Fig. 4:**
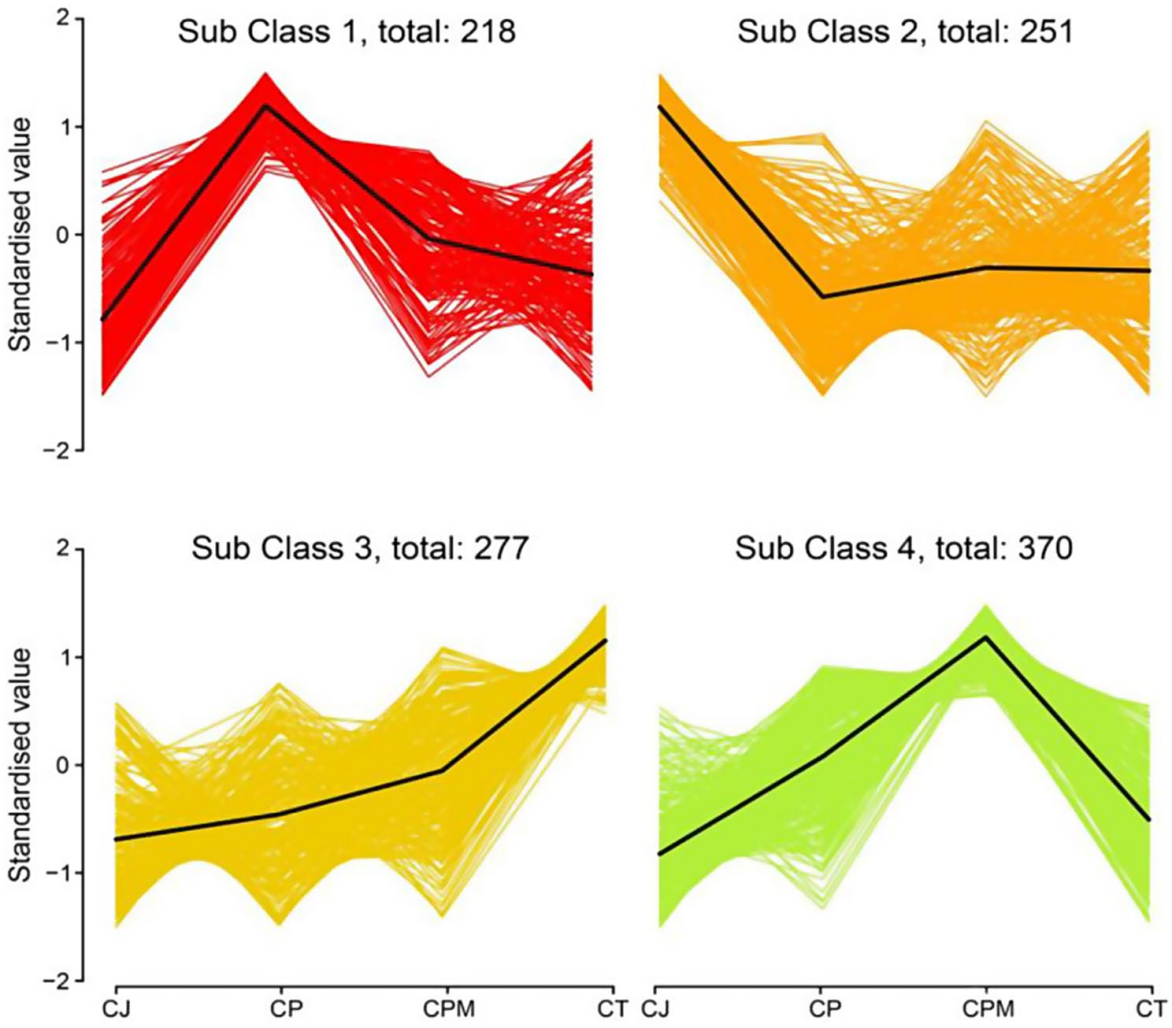
*K*-means clustering analysis of the patterns of 1,116 differential metabolites in Codonopsis Radix and *Campanumoea javanica* Bl

*K*-means cluster analysis was applied to a better understanding of the relative content trend of metabolites in distinct species. Approximately 1,116 metabolites were divided into four subclasses (Fig. 4). In sub-class 1, 218 compounds (19.53%) were relatively high in CP, mainly Lipids (17.43%), Phenolic acids (15.60%), and Flavonoids (12.84%). In sub-class 2, 251 compounds (22.49%) were relatively high in CJ, the majority of which were phenolic acids (26.29%), lipids (19.12%), and flavonoids (13.15%). In sub-class 3, 277 compounds (24.82%) were relatively high in CT, mainly phenolic acids (20.94%), amino acids and derivatives (14.08%), and alkaloids (13.36%). In sub-class 4, 370 compounds (33.15%) were relatively high in CPM, the majority of which were phenolic acids (17.13%), organic acids (16.57%), and saccharides (15.17%). Metabolic pathway annotation was performed by the KEGG database for 251 compounds enriched in CJ, and a total of 67 metabolic pathways were enriched, which were mainly enriched in Metabolic pathways (ko01100), Biosynthesis of secondary metabolites (ko01110), ABC Transporters (ko02010), and Biosynthesis of amino acids (ko01230).

#### Screening for differential metabolites

**Fig. 5:**
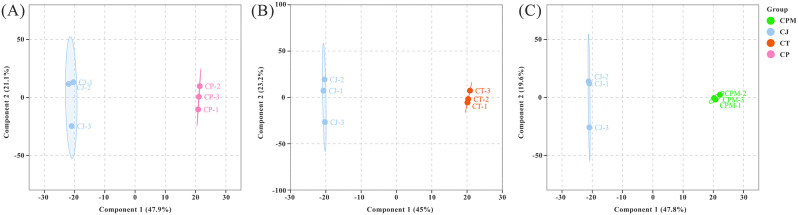
OPLS-DA score chart. A: CP vs. CJ; B: CT vs. CJ; C: CPM vs. CJ

**Fig. 6:**
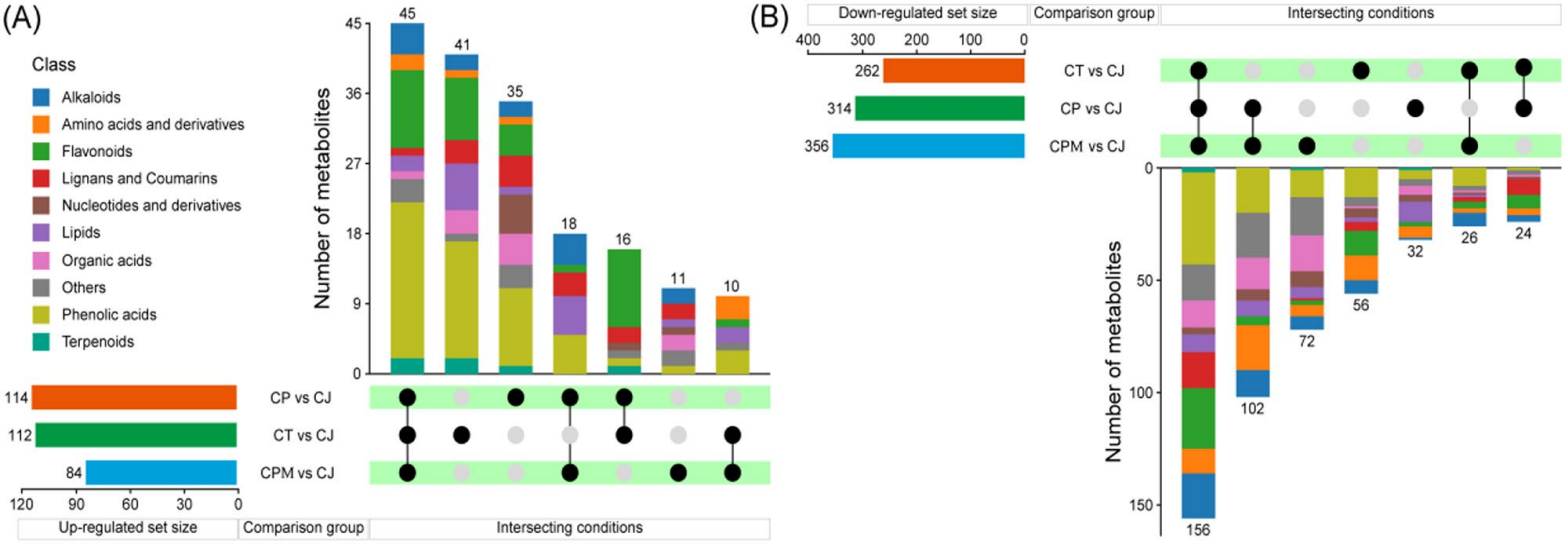
The specific expressions of DMs (**A**) Up-regulated differential metabolites in comparison groups; (**B**) Down-regulated differential metabolites in comparison groups

To screen for differential metabolites, we modeled the comparison of CJ with CPM, CT, and CP by OPLS-DA, and the Q^2^ values were all greater than 0.90, which is an excellent model (Fig. 5A and B, and 5C). The screening of differential metabolites were conducted using criteria of | log_2_(fold change) |>1 and VIP ≥ 1. CJ was compared with CP, CPM, and CT in a two-by-two comparison to obtain the following three comparison groups of differential metabolites: CP vs. CJ, CPM vs. CJ, and CT vs. CJ. The specific expressions of DMs are illustrated in Fig. 6.

Compared with CP, CT, and CPM, 114,112 and 84 DMs were up-regulated in CJ (Fig. 6A), but 314, 262 and 356 DMs were down-regulated in CJ (Fig. 6B). We discovered that there were 35, 11, and 41 DMs up-regulated only in CP vs. CJ, CPM vs. CJ, and CT vs. CJ, respectively, and 32, 72, and 56 DMs were down-regulated, respectively. 45 DMs were all up-regulated in CP vs. CJ, CPM vs. CJ, and CT vs. CJ, including phenolic acids (20 DMs), flavonoids (10 DMs), alkaloids (4 DMs), others (3 DMs), amino acids and derivatives (2 DMs), lipids (2 DMs), terpenoids (2 DMs), lignans and coumarins (1 DM), and organic acids (1 DM).

#### Screening for dominant differential metabolites

**Fig. 7:**
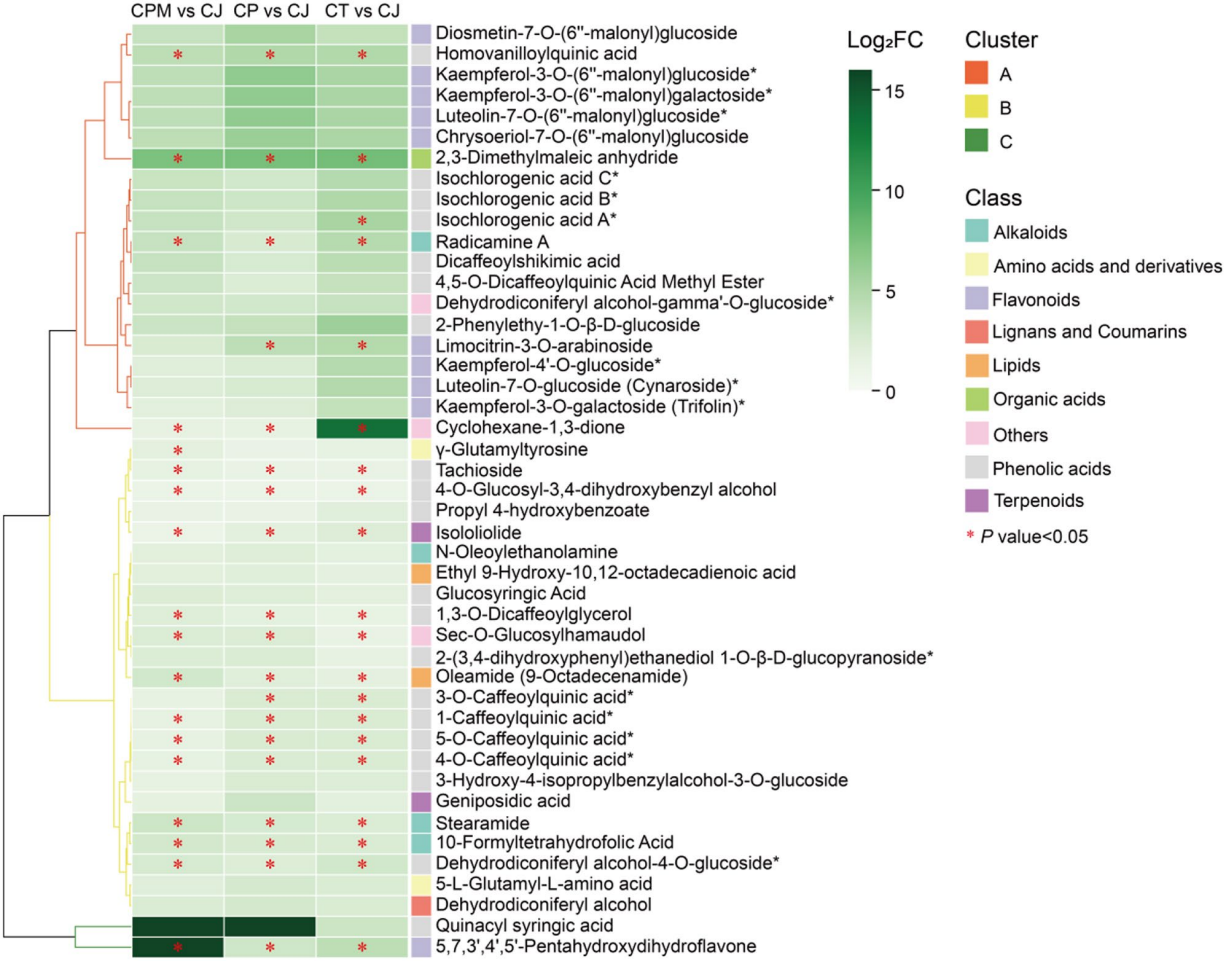
Hierarchical clustering heat map of *Campanumoea javanica* Bl. dominant differential metabolites

Based on analysis of metabolite difference, the dominant differential metabolites in CJ were identified by screening for up-regulated DMs and those with a significance level (*P* < 0.05) in CJ. Total of 17 dominant differential metabolites were found in CJ (Fig. 7). Among them, 12 components’ Canonical SMILES were retrieved in the PubChem database, including 1-caffeoylquinic acid*, 1,3-O-dicaffeoylglycerol, tachioside, 4-O-caffeoylquinic acid*, radicamine A, stearamide, 10- formyltetrahydrofolic acid, oleamide (9-octadecenamide), 2,3-dimethylmaleic anhydride, isololiolide, sec-O-glucosylhamaudol, and cyclohexane-1,3-dione.

### Network pharmacological analysis of CJ dominant differential metabolites

**Fig. 8:**
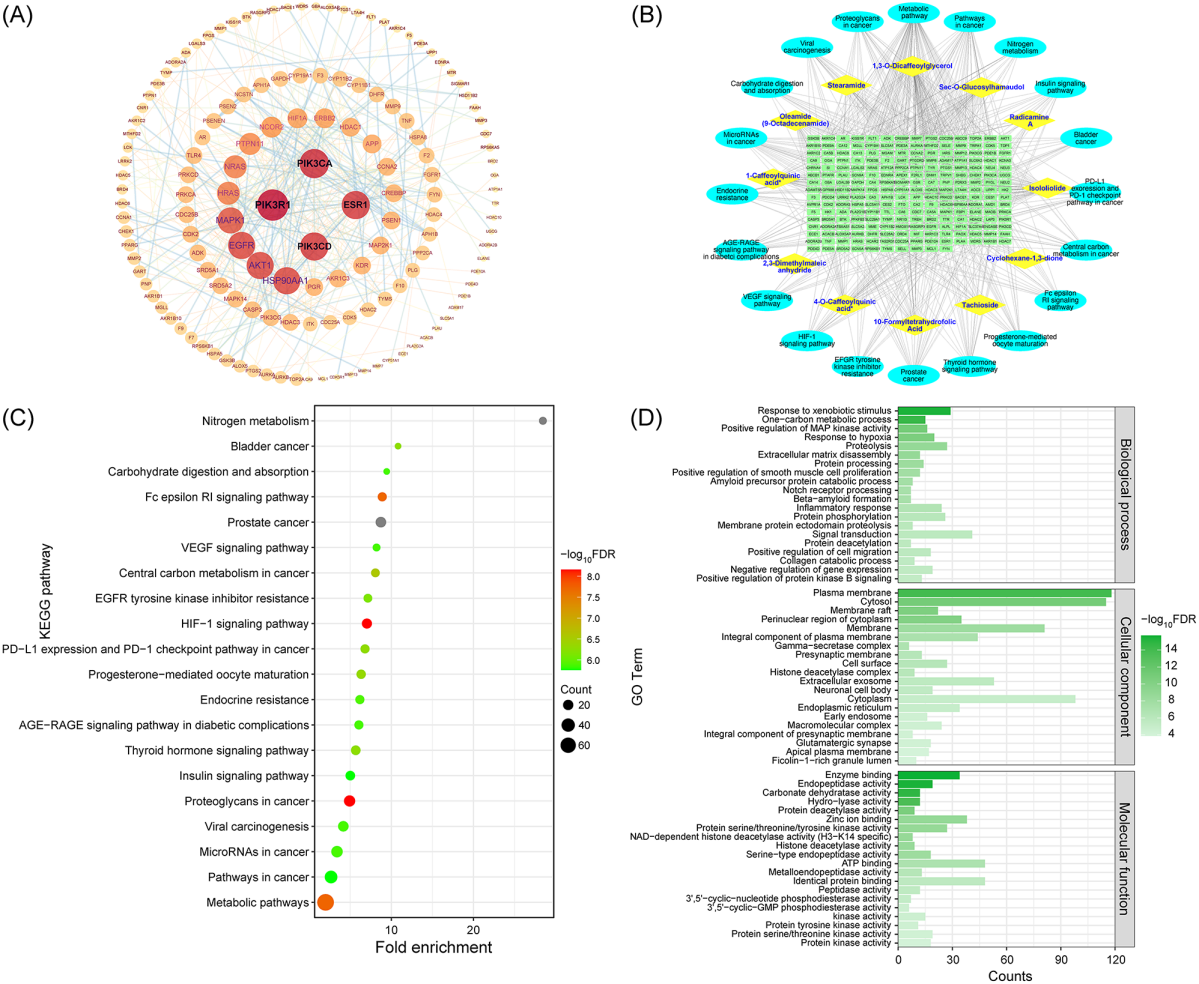
(**A**) protein-protein interaction network diagram of *Campanumoea javanica* Bl.; (**B**) *Campanumoea javanica* Bl. dominant chemical composition-target-pathways network; (**C**) KEGG analysis of signaling pathways enriched by candidate genes; (**D**) GO analysis of candidate targets, including molecular function, cellular component, and biological processes

The corresponding target proteins for the twelve dominant differential metabolites in CJ were estimated, leading to the identification of 229 targets. The protein-protein interaction (PPI) network was constructed with 229 targets using Cytoscape 3.9.1 (Fig. 8A). The targets with ≥ 17 degrees were PIK3R1, PIK3CA, PIK3CD, ESR1, HSP90AA1, AKT1, EGFR, suggesting a more crucial function for the targets in the PPI network map.

On 229 targets, GO functional enrichment analysis, KEGG pathway enrichment analysis, and disease enrichment analysis were performed utilizing the David database (6.8, https://david.ncifcrf.gov/). The statistical significance threshold of *P* < 0.01 was employed for the GO functional analysis, KEGG pathway enrichment analysis, and disease enrichment analysis. Approximately 873 items in total, including 589 biological processes (BP), 97 cell compositions (CC), 187 molecular functions (MF), 147 KEGG pathway analysis entries, and six disease enrichment analysis entries, were derived from the GO functional analysis. Visualization of the top 20 entries for each category was performed via the bioinformatics website. (Figure 8C and D, http://www.bioinformatics.com.cn/). The composition of BP primarily consists of xenobiotic stimuli, a one-carbon metabolic process, and positive regulation of MAP kinase activity. CC primarily included plasma membrane, cytosol, and membrane raft. Enzyme binding, endopeptidase activity, and carbonate dehydratase activity were MF’s primary inclusions in the GO functional analysis. Nitrogen metabolism, cancer (prostate cancer, proteoglycans in cancer, bladder cancer), and signaling pathway (HIF-1 signaling pathway, Fc epsilon RI signaling pathway, thyroid hormone signaling pathway) were among the major topics covered by KEGG pathway analysis. Using Cytoscape software (3.7.0), a “drug-component-target-network” map of CJ was generated based on the evaluation of 12 components, 229 targets, and 20 pathways (Fig. 8B).

### Molecular docking of core targets and dominant differential metabolites

**Fig. 9:**
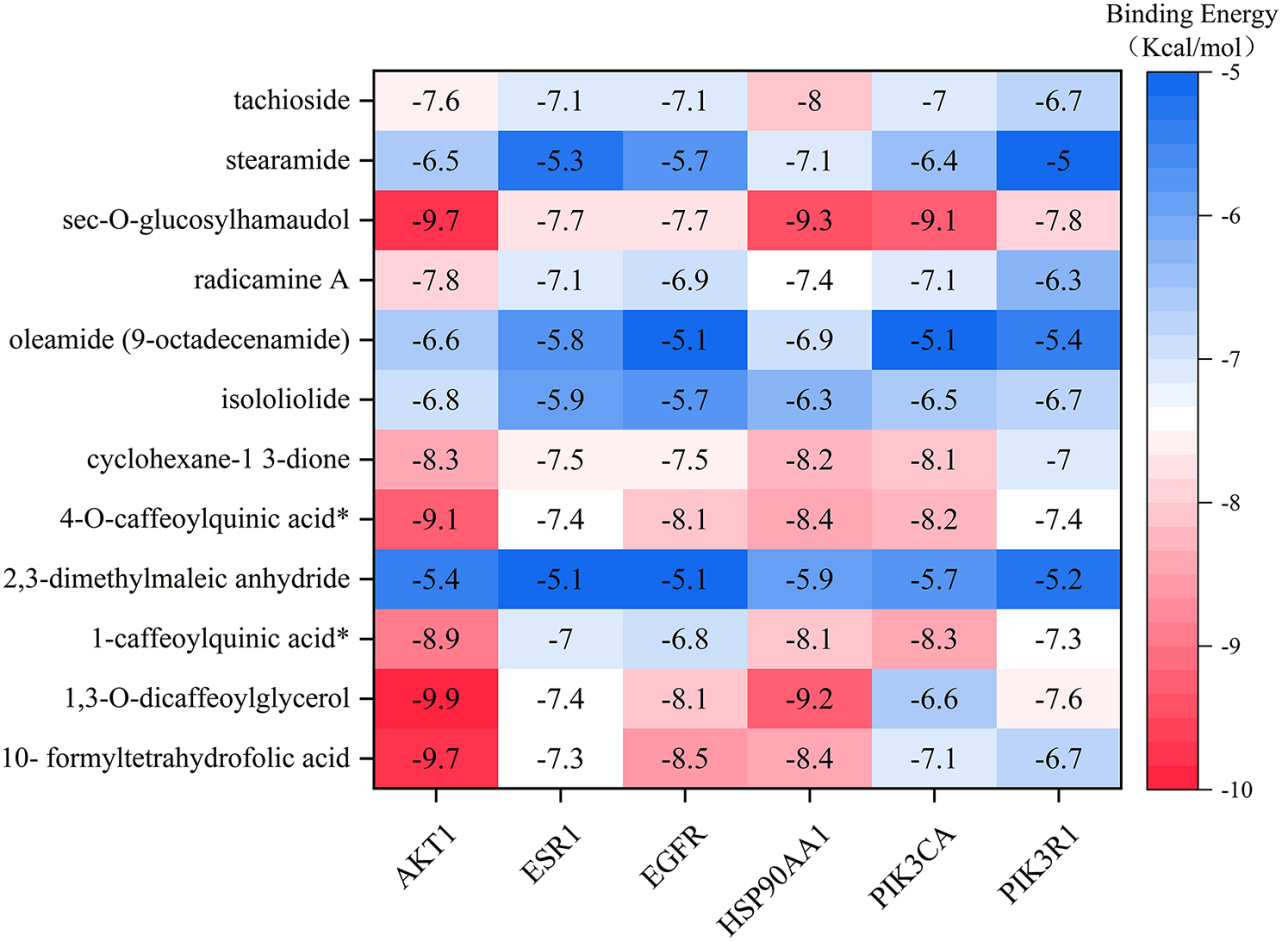
Molecular docking heat map of the dominant differential metabolites of *Campanumoea javanica* Bl. with key targets

Based on the criteria of top 5 Degree values and topological parameters greater than the median (HSP90AA1, AKT1, EGFR had the Same Degree value and 4 topological parameters of PIK3CD were below the median), 6 target proteins were finally selected (PIK3R1, PIK3CA, ESR1, AKT1, HSP90AA1, EGFR) and molecularly docked with 12 dominant metabolites. 72 receptor-ligand docking results were finally obtained, as shown in Fig. 9.

**Fig. 10:**
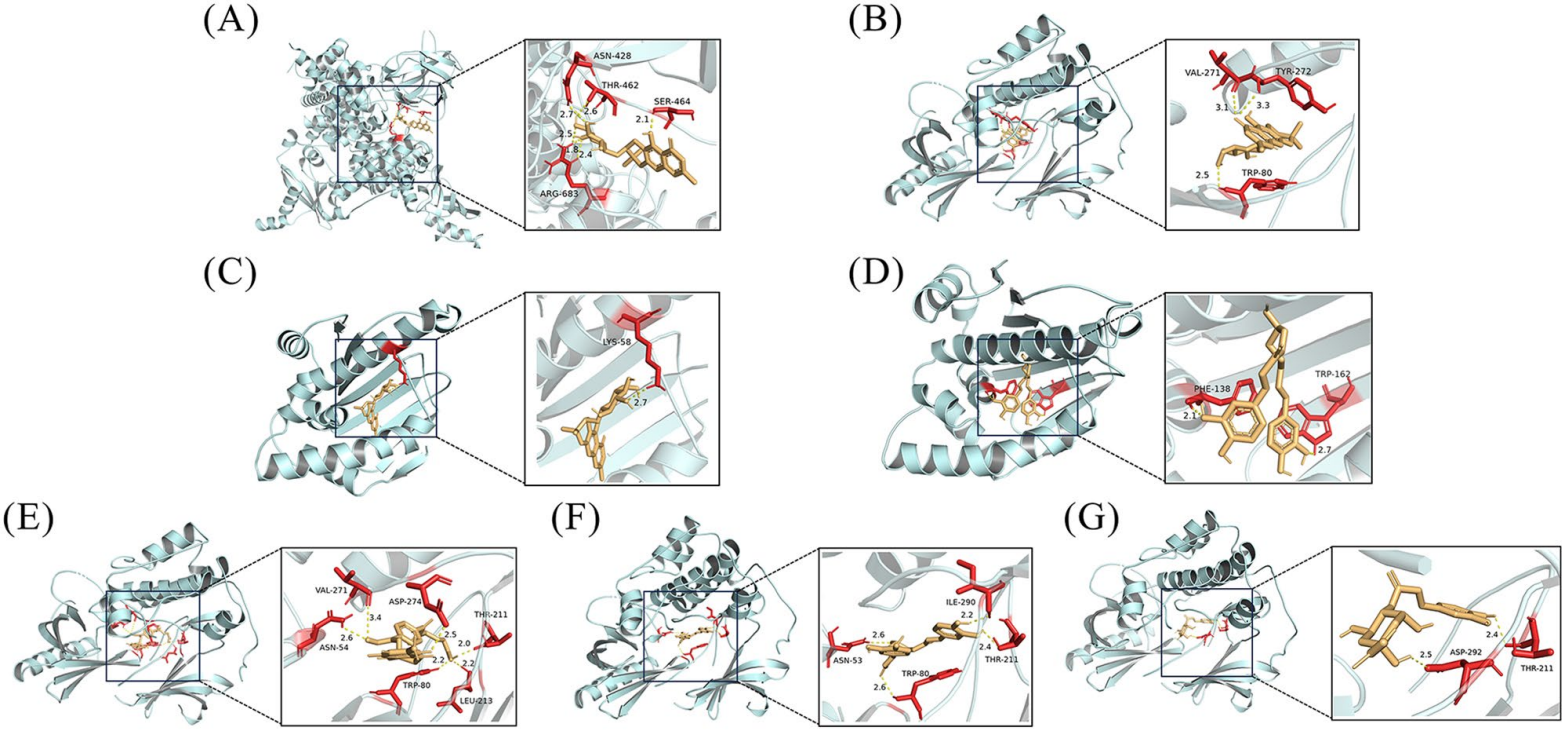
Visualization diagram of molecular docking. (A: PIK3CA ~ sec-O-glucosylhamaudol; B: AKT1 ~ sec-O-glucosylhamaudol; C: HSP90AA1 ~ sec-O-glucosylhamaudol; D: HSP90AA1 ~ 1,3-O-dicaffeoylglycerol; E: AKT1 ~ 10-formyltetrahydrofolic acid; F: AKT1 ~ 1,3-O-dicaffeoylglycerol; G: AKT1 ~ 4-O-caffeoylquinic acid*)

The binding energies of the 72 receptor-ligand pairs were all ≤ -5.0 kcal/mol, with 7 pairs having energies ≤ -9.0 kcal/mol, accounting for 9.7%. This indicates a strong binding affinity between the predicted active ingredients and key targets in this study. Finally, the top 7 docking results based on molecular docking scores were visualized using PyMol 2.4.1 software, as shown in Fig. 10.

## Discussion

### Differences between Campanumoea javanica bl. and Codonopsis Radix

The metabolite analysis results showed that 1038 (93.01%) shared metabolites were identified in CJ, CPM, CP, and CT, and 251 compounds (22.49%) in CJ had content dominance, indicating that CJ and CR metabolite species were highly similar, but with significant differences in content. In addition, CPM, CP, and CT had 1073 (96.15%) shared metabolites, and the cluster analysis clustered them into one group, indicating their metabolite profiles were almost identical. This experiment greatly enhanced the metabolic database of CR compared to Tang’s study [31]. The above evidence confirmed the correctness of the Chinese Pharmacopoeia in treating CPM, CP, and CT all as sources of CR. It was consistent with the “similar species, similar effects” hypothesis advanced by Xie [32]. Subsequently, on the basis of this experiment, in-depth research can be conducted to verify the scientific basis of the folk use of CJ as a CR substitute. There are many successful cases have been obtained by applying the same family or genus of plants to discover new drug sources. For example, reserpine, an active ingredient in the treatment of hypertension, was originally only obtained from the *Rauvolfia serpentine*. In recent years, researchers have isolated and identified it in other plant from the same genus [33].

### Great potential for food exploitation of Campanumoea javanica bl

Our research found that CJ was rich in terpenoids, flavonoids, and lipids, with the relative content of 251 compounds including 66 phenolic acids, 48 lipids, and 33 flavonoids was higher than that of CR. Commonly employed as biological preservatives in food, phenolic acids function primarily to prevent oxidative damage by acting as antioxidants. Additionally, they predominantly contribute to the enhancement of overall health through their antimicrobial and anti-inflammatory properties [34]. Terpenoids are the most prevalent class of chemicals in natural products and extensively dispersed in nature, as a significant class of secondary metabolites in plants. They are widely used as raw materials in pharmaceuticals, food, and cosmetics, as well as taking part in physiological processes like plant growth and development and environmental response. In the future, they may also be used as natural additive, such as essence, sweeteners, nutrition enhancers [35]. Flavonoids are necessary nutrients that are crucial to health care but cannot be produced by the human body and must instead be consumed from outside sources. Daily consumption of fruits and vegetables high in flavonoids can significantly lower the risk of developing cancer. Foods that are high in these CJ components will be developed into healthy congee, savory dishes, cold dishes, etc., not only to meet the needs of the public’s healthcare and broaden the range of uses for CJ but also to increase the income of the farmers in the producing areas [36, 37]. One of the nutrients in a living thing is called lipid, which also acts as a carbon and energy reserve for the body as well as a mediator of cellular signaling pathways and a stress regulator, among other functions. Lipids are also an essential part of the biofilm. The flavor and nutritional value of foods that are lipid-rich are directly correlated with their lipid content and composition [38]. To conduct application and processing research on these CJ plant resources, CJ can thus draw on the existing application forms and processing methods of CR. This helps to increase the success rate of new product development for CJ resources, lower the risk of product development, and thus promote the in-depth development and application of CJ plant resources.

### Campanumoea javanica bl. Has the potential for new drug exploitation

Polyethylenimine can be modified by the dominant metabolite 2,3-dimethylmaleic anhydride from CJ to improve the antitumor effects of conventional therapy [39], whereas isooliolide changes the expression of proteins that are crucial for the apoptotic cascade and have antitumor properties [40]. The 12 major dominant differential metabolite components of CJ were also predicted as targets using the network pharmacology method, and the results revealed that they primarily involved 229 targets, including PIK3CA, PIK3R1, HSP90AA1, EGFR, MAPK1, etc., and the targets were enriched in 147 pathways, including Nitrogen metabolism, Prostate cancer, HIF-1 signaling pathway, and proteoglycans in cancer. Sun et al. discovered that PIK3CA is responsible for encoding the catalytic subunit p110a protein and that its release and activation can keep PI3K in a continuously active state. Additionally, changes in PIK3CA can induce other downstream PI3K kinases, which can activate the entire pathway and produce a pro-inflammatory effect [41]. Wu et al. discovered that PIK3R1 was a negative regulator of PI3K/Akt/mTOR signaling and that lipopolysaccharide can downregulate its expression and contribute to the progression of inflammation [42]. Wang et al. discovered that HSP90AA1 is a related gene that promotes the progression of squamous carcinoma in the lung [43]. Jin et al. discovered that EGFR has rapid endocytosis activity and is expressed in numerous solid tumors, and Li et al. discovered that prolactin targets are MAPK1 and EGFR, etc. [44, 45]. As a result, CJ is thought to perform prominently in treating inflammation, cancer, breastfeeding. Based on network pharmacology and molecular docking, computer simulations were used to predict and validate the potential mechanism of action of the major metabolites of CJ. Subsequent ex vivo and in vivo efficacy experiments are needed to validate and provide theoretical support for the clinical application of CJ.

## Conclusions

In this study, we employed metabolomics, network pharmacology, and molecular docking verification to delve into the metabolites of CJ and CR and predict the pharmacodynamics of the dominant differential metabolites of CJ. The results of the metabolomic analysis showed an average of 1061 (95.07%) shared metabolites in CJ and CRs but with remarkably different metabolite profiles. CJ had a higher relative concentration of 251 components, including 66 phenolic acids, 48 lipids, and 33 flavonoids compared to CRs. These components indicated that CJ possessed good edible characteristics and food development value. Furthermore, the dominant differential metabolites of CJ have shown medical utility in treating cancer, inflammation, and lactation, indicating its value for new drug development. However, further exploration is required to understand the specific mechanism of the different components and pharmacological effects due to the lack of consideration of the relative content differences and interactions between the components. This research has provided initial insights into the metabolite differentiation between CJ and CR and the dominant metabolites from the perspective of plant metabolomics. It also provides a conceptual framework for CJ’s advancement of novel pharmaceuticals and significantly improves the metabolite databases on CJ and CR.

## Data Availability

The data supporting the fndings of this study are available from the corresponding author, W.F. (wufaming@zmu.edu.cn), upon request.

## Abbreviations

CJ: Campanumoea javanica Bl
CPM: Codonopsis pilosula var. *modesta* (Nannf.) L.T. Shen
CP: Codonopsis pilosula (Franch.) Nannf
CT: Codonopsis tangshen Oliv
CR: Codonopsis Radix
RT: Retention time
GO: Gene Ontology
BP: Biological processes
CC: Cell compositions
MF: Molecular functions
ESI: Electrospray ionization
PPI: Protein-protein interaction
MRM: Multiple reaction monitoring
DMs: Differential metabolites
VIP: Variable importance in projection
MWDB: Metware database
KEGG: Kyoto Encyclopedia of Genes and Genomes
OPLS-DA: Orthogonal partial least squares-discriminant analysis
UPLC-ESI-MS/MS: Ultra-performance liquid chromatography-electrospray ionization-tandem mass spectrometry
